# Supplementary material for: Antioxidant Effects of Exogenous Mitochondria: The Role of Outer Membrane Integrity
Source: Antioxidants (Basel). 2025 Aug 2;14(8):951. doi: 10.3390/antiox14080951 (PMC12382848; doi:10.3390/antiox14080951)
Supplement: Supplementary file 1 [file antioxidants-14-00951-s001.zip › antioxidants-3719941-supplementary.pptx]

## Slide 1
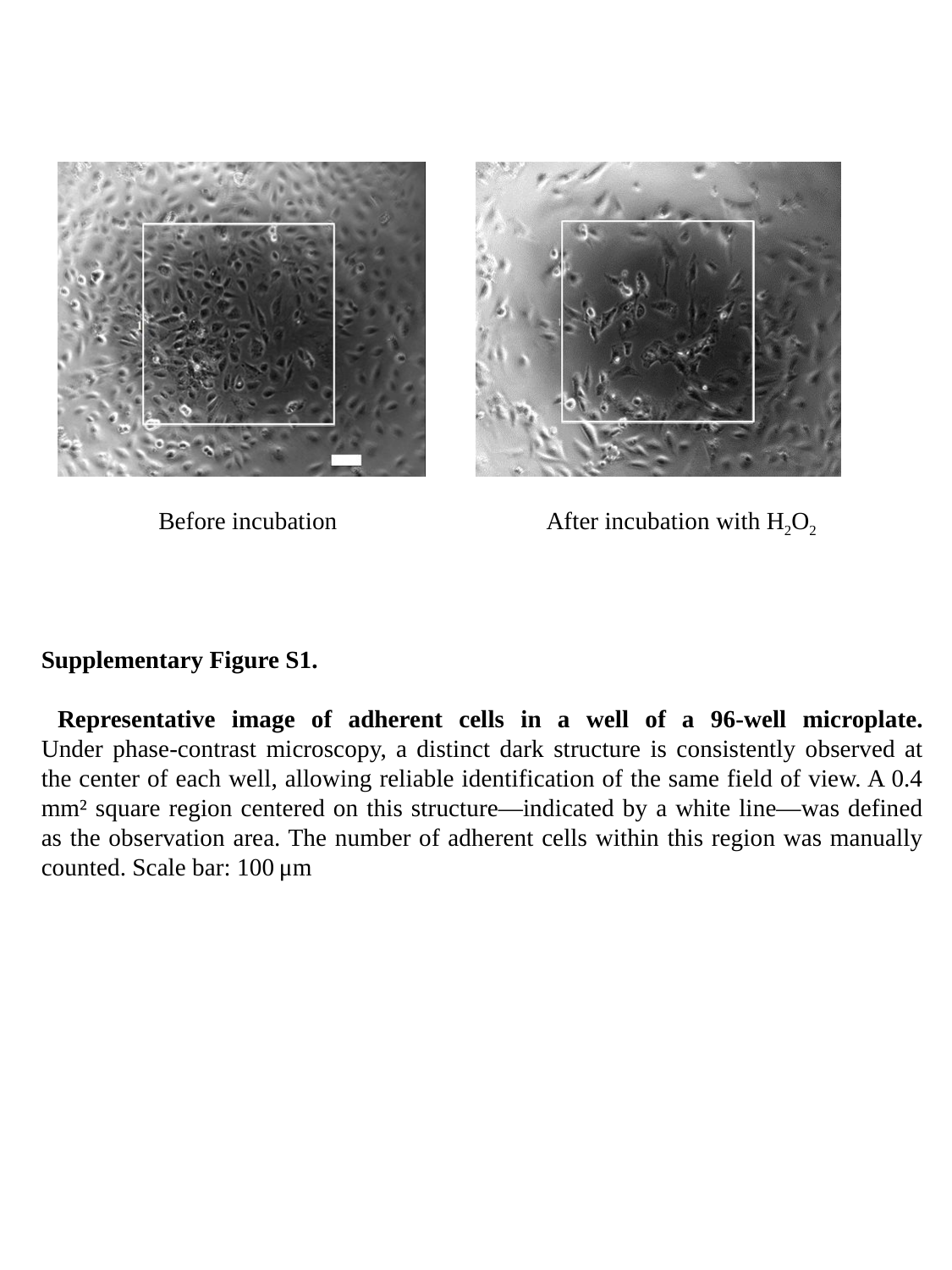

Before incubation
After incubation with H2O2
Supplementary Figure S1.
 Representative image of adherent cells in a well of a 96-well microplate.
Under phase-contrast microscopy, a distinct dark structure is consistently observed at the center of each well, allowing reliable identification of the same field of view. A 0.4 mm² square region centered on this structure—indicated by a white line—was defined as the observation area. The number of adherent cells within this region was manually counted. Scale bar: 100 μm

## Slide 2
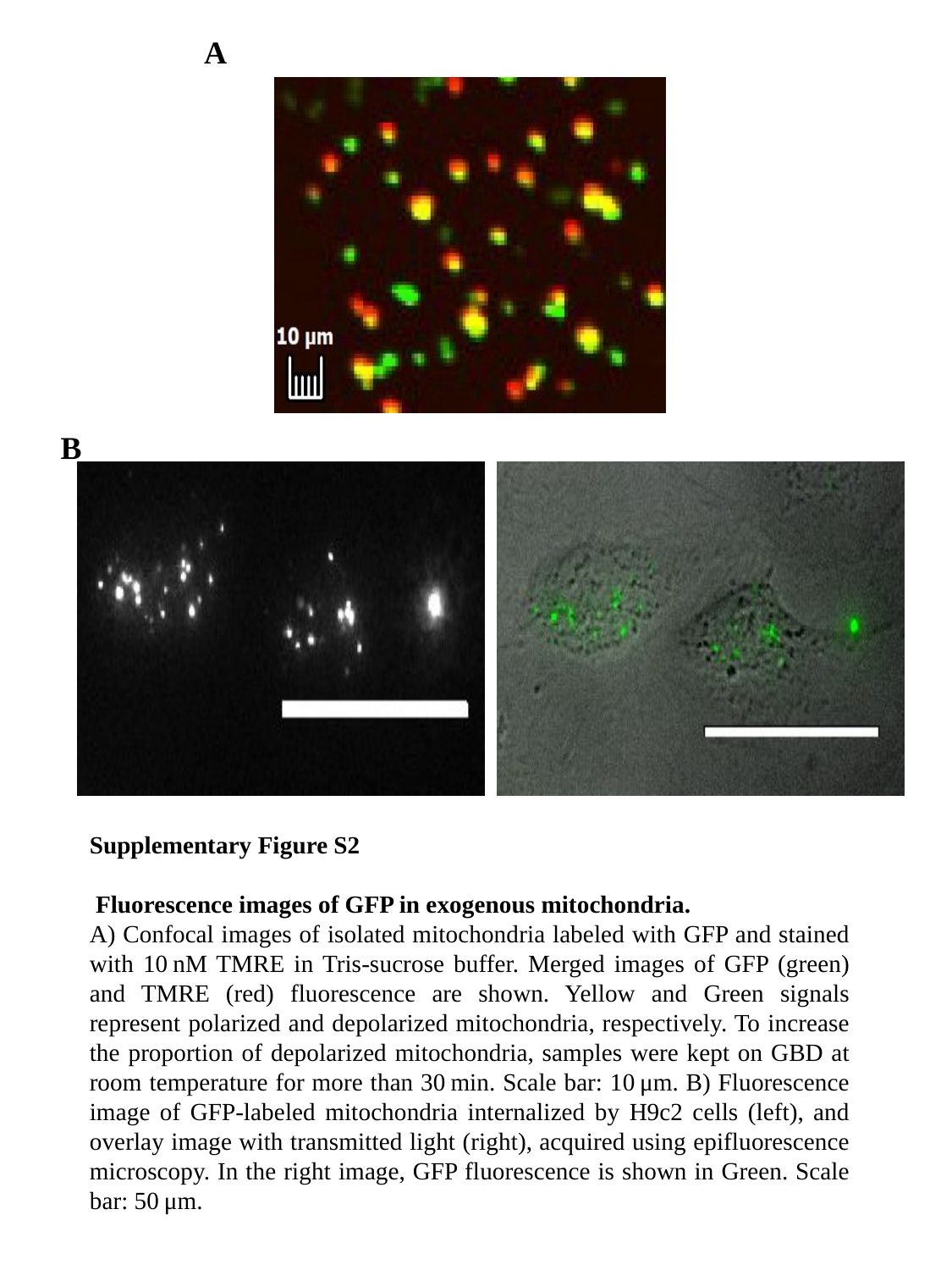

A
B
Supplementary Figure S2
 Fluorescence images of GFP in exogenous mitochondria.
A) Confocal images of isolated mitochondria labeled with GFP and stained with 10 nM TMRE in Tris-sucrose buffer. Merged images of GFP (green) and TMRE (red) fluorescence are shown. Yellow and Green signals represent polarized and depolarized mitochondria, respectively. To increase the proportion of depolarized mitochondria, samples were kept on GBD at room temperature for more than 30 min. Scale bar: 10 μm. B) Fluorescence image of GFP-labeled mitochondria internalized by H9c2 cells (left), and overlay image with transmitted light (right), acquired using epifluorescence microscopy. In the right image, GFP fluorescence is shown in Green. Scale bar: 50 μm.

## Slide 3
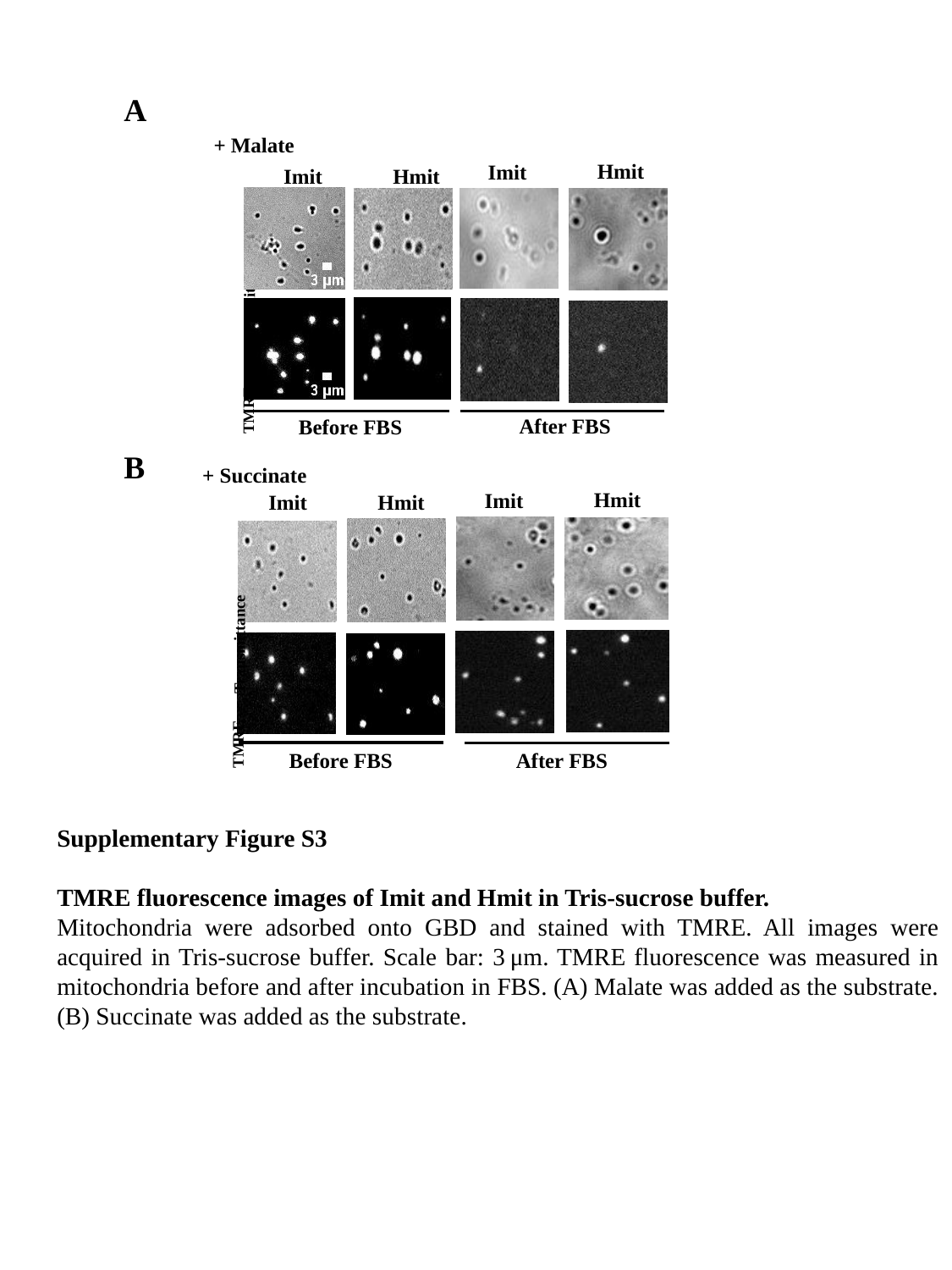

A
+ Malate
Hmit
Imit
Hmit
Imit
	Transmittance
	TMRE
 After FBS
Before FBS
B
 + Succinate
Hmit
Imit
Hmit
Imit
	Transmittance
	TMRE
 After FBS
Before FBS
Supplementary Figure S3
TMRE fluorescence images of Imit and Hmit in Tris-sucrose buffer.
Mitochondria were adsorbed onto GBD and stained with TMRE. All images were acquired in Tris-sucrose buffer. Scale bar: 3 μm. TMRE fluorescence was measured in mitochondria before and after incubation in FBS. (A) Malate was added as the substrate. (B) Succinate was added as the substrate.

## Slide 4
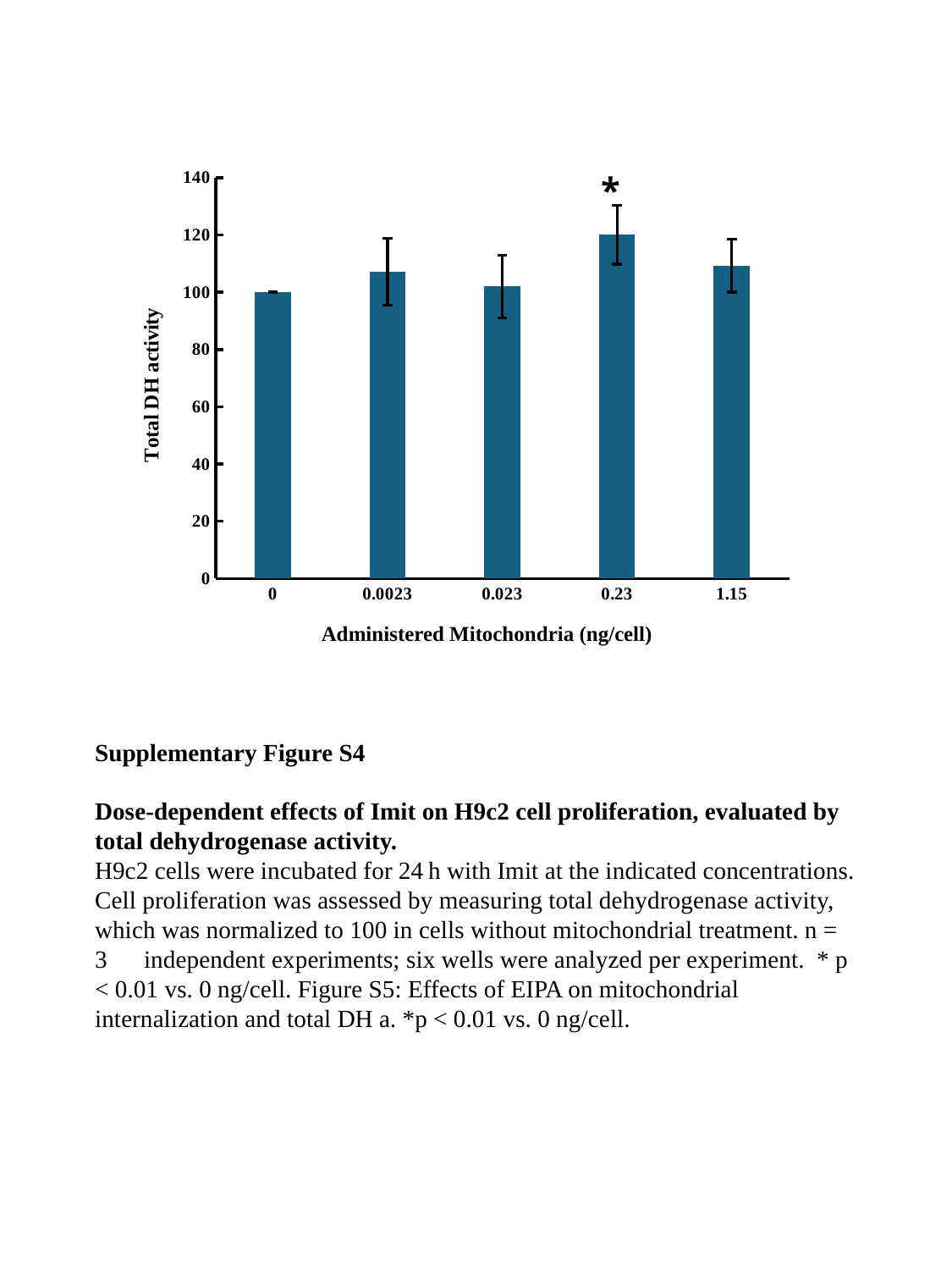

### Chart
| Category | |
|---|---|
| 0 | 100.0000147589033 |
| 2.3E-3 | 107.06092201189213 |
| 2.3E-2 | 101.96463479354975 |
| 0.23 | 120.03449744643544 |
| 1.1499999999999999 | 109.18943580651941 |Administered Mitochondria (ng/cell)
Supplementary Figure S4
Dose-dependent effects of Imit on H9c2 cell proliferation, evaluated by total dehydrogenase activity.
H9c2 cells were incubated for 24 h with Imit at the indicated concentrations. Cell proliferation was assessed by measuring total dehydrogenase activity, which was normalized to 100 in cells without mitochondrial treatment. n = 3　independent experiments; six wells were analyzed per experiment. * p < 0.01 vs. 0 ng/cell. Figure S5: Effects of EIPA on mitochondrial internalization and total DH a. *p < 0.01 vs. 0 ng/cell.

## Slide 5
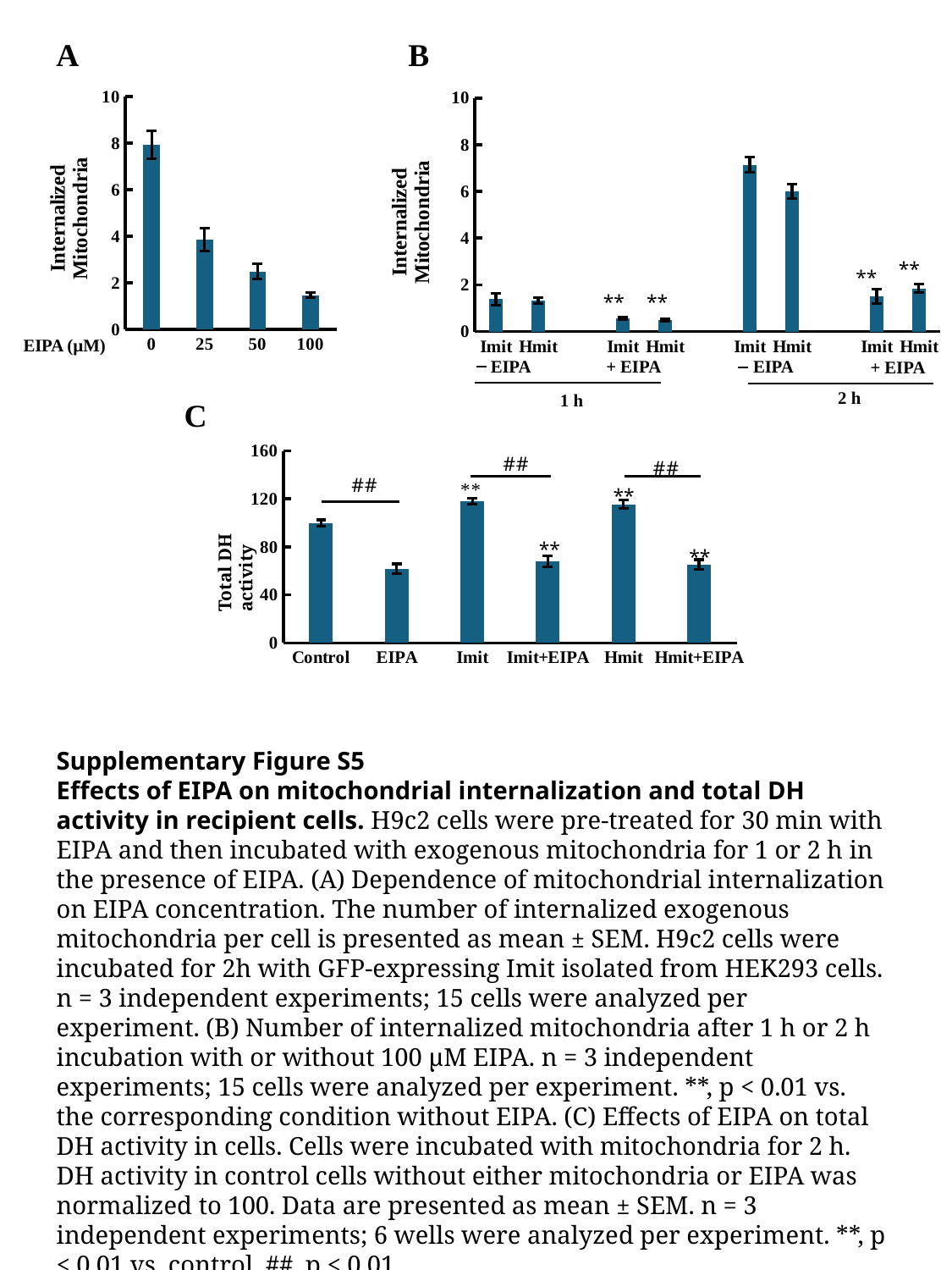

A
B
### Chart
| Category | |
|---|---|
| Imit | 1.3888159687338837 |
| Hmit | 1.326195483268055 |
| | None |
| Imit | 0.5696859293979673 |
| Hmit | 0.4908427242309362 |
| | None |
| Imit | 7.1435949978510465 |
| Hmit | 5.997579924075232 |
| | None |
| Imit | 1.4984410623982067 |
| Hmit | 1.83952398205 |
### Chart
| Category | |
|---|---|
| 0 | 7.9300071218705455 |
| 25 | 3.8555528996000747 |
| 50 | 2.4830173394776787 |
| 100 | 1.466261154532044 |EIPA (µM)
EIPA
+ EIPA
EIPA
+ EIPA
2 h
1 h
C
### Chart
| Category | |
|---|---|
| Control | 99.99657298805089 |
| EIPA | 61.72593558116043 |
| Imit | 118.1341056802715 |
| Imit+EIPA | 67.93061939081413 |
| Hmit | 115.47150379308765 |
| Hmit+EIPA | 65.35974285364772 |Total DH activity
Supplementary Figure S5
Effects of EIPA on mitochondrial internalization and total DH activity in recipient cells. H9c2 cells were pre-treated for 30 min with EIPA and then incubated with exogenous mitochondria for 1 or 2 h in the presence of EIPA. (A) Dependence of mitochondrial internalization on EIPA concentration. The number of internalized exogenous mitochondria per cell is presented as mean ± SEM. H9c2 cells were incubated for 2h with GFP-expressing Imit isolated from HEK293 cells. n = 3 independent experiments; 15 cells were analyzed per experiment. (B) Number of internalized mitochondria after 1 h or 2 h incubation with or without 100 μM EIPA. n = 3 independent experiments; 15 cells were analyzed per experiment. **, p < 0.01 vs. the corresponding condition without EIPA. (C) Effects of EIPA on total DH activity in cells. Cells were incubated with mitochondria for 2 h. DH activity in control cells without either mitochondria or EIPA was normalized to 100. Data are presented as mean ± SEM. n = 3 independent experiments; 6 wells were analyzed per experiment. **, p < 0.01 vs. control. ##, p < 0.01.

## Slide 6
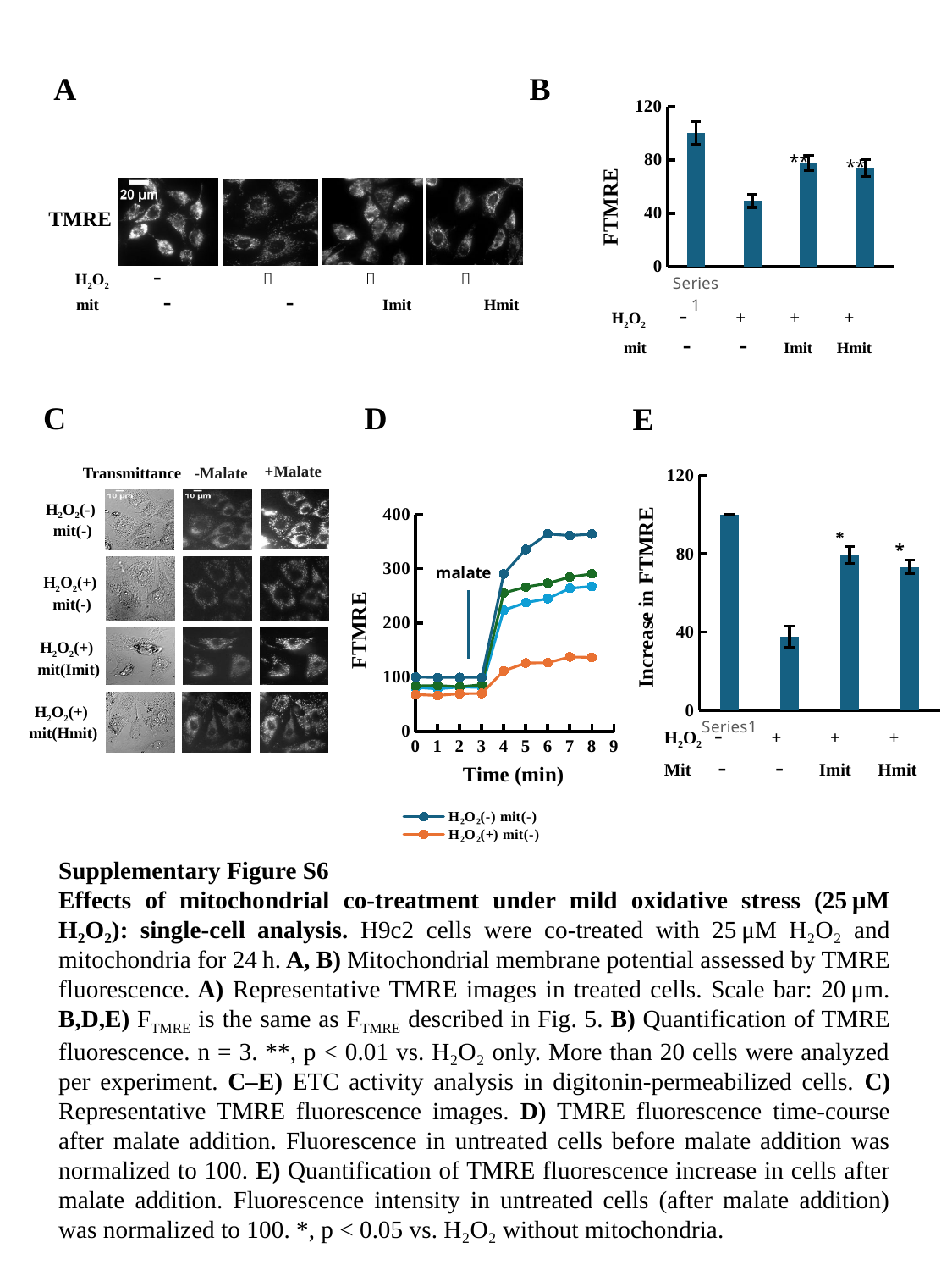

B
A
### Chart
| Category | |
|---|---|
| | 99.9999952144323 |
| | 49.154130921651685 |
| | 77.59396091877201 |
| | 73.79281965759823 |H2O2 - + + +
mit - - Imit Hmit
**
**
TMRE
H2O2 - 　　 ＋　　　　 ＋　　 　 ＋
 mit 　 -　 - Imit Hmit
C
D
E
+Malate
Transmittance
-Malate
H2O2(-)
mit(-)
H2O2(+)
mit(-)
H2O2(+)
mit(Imit)
H2O2(+)
mit(Hmit)
### Chart
| Category | |
|---|---|
| | 99.99999039779684 |
| | 37.7353778101101 |
| | 79.3382640926259 |
| | 73.2802017440205 |H2O2 - + + +
Mit - - Imit Hmit
### Chart
| Category | H₂O₂(-) mit(-) | H₂O₂(+) mit(-) | H₂O₂(+) mit(Imit) | H₂O₂(+) mit(Hmit) |
|---|---|---|---|---|Time (min)
Supplementary Figure S6
Effects of mitochondrial co-treatment under mild oxidative stress (25 μM H₂O₂): single-cell analysis. H9c2 cells were co-treated with 25 μM H₂O₂ and mitochondria for 24 h. A, B) Mitochondrial membrane potential assessed by TMRE fluorescence. A) Representative TMRE images in treated cells. Scale bar: 20 μm. B,D,E) FTMRE is the same as FTMRE described in Fig. 5. B) Quantification of TMRE fluorescence. n = 3. **, p < 0.01 vs. H₂O₂ only. More than 20 cells were analyzed per experiment. C–E) ETC activity analysis in digitonin-permeabilized cells. C) Representative TMRE fluorescence images. D) TMRE fluorescence time-course after malate addition. Fluorescence in untreated cells before malate addition was normalized to 100. E) Quantification of TMRE fluorescence increase in cells after malate addition. Fluorescence intensity in untreated cells (after malate addition) was normalized to 100. *, p < 0.05 vs. H₂O₂ without mitochondria.
